# Correction: Success-efficient/failure-safe strategy for hierarchical reinforcement motor learning

**DOI:** 10.1371/journal.pcbi.1013537

**Published:** 2025-09-30

**Authors:** The PLOS Computational Biology Staff

S4 Fig was uploaded incorrectly. Please see the correct S4 Fig here.

## Supporting information

S4 Fig Trials at which adaptation measures reached plateaus. Trial numbers and standard deviations of when the Number of Failed Trials, Trajectory Area, Initial Trajectory Area, Co-Contraction, and Smoothness reached their respective plateaus.
(PDF)

The publisher apologizes for the error.
