# Supplementary figures and images for: Success-efficient/failure-safe strategy for hierarchical reinforcement motor learning
Source: PLoS Comput Biol. 2025 May 9;21(5):e1013089. doi: 10.1371/journal.pcbi.1013089 (PMC12121909; doi:10.1371/journal.pcbi.1013089)

Position  $y$  / m

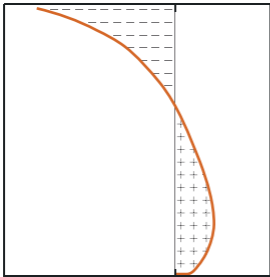

Position  $x$  / m

Position  $y$  / m

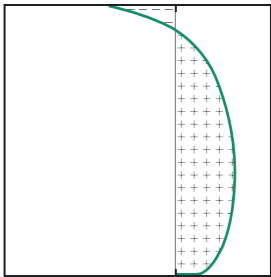

Position  $x$  / m

Supplement: S1 Fig — Illustration of how Trajectory Area is computed as the integral of the deviation from a straight-line reference in the sagittal plane. The hatched regions represent the calculated area where regions of negative area are hatched with minus signs (–), and regions of positive area are hatched with plus signs (+). (PDF) [file pcbi.1013089.s001.pdf]

A

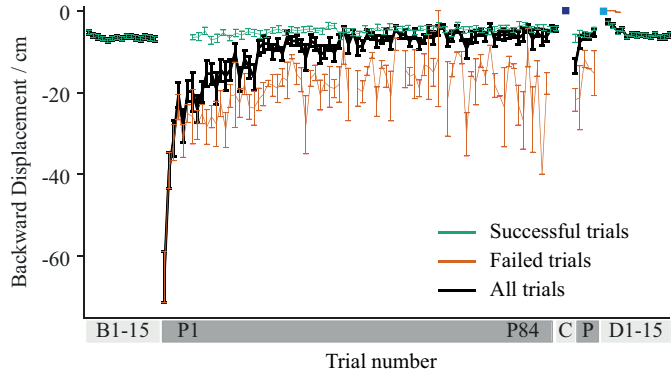

B

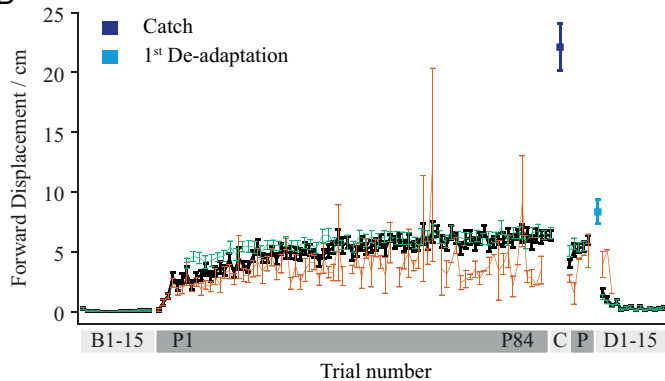

Supplement: S2 Fig — Same as Fig 1D and 1E except that the measure is Forward Peak Displacement (A) and Backward Peak Displacement (B). (PDF) [file pcbi.1013089.s002.pdf]

A

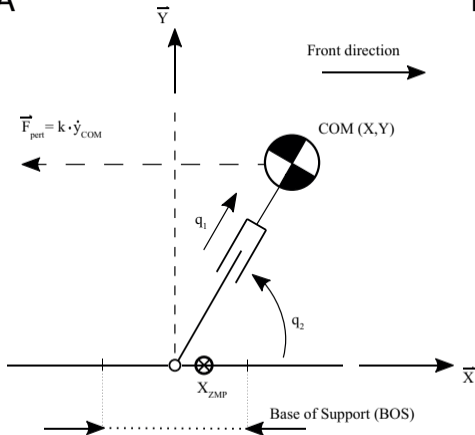

B

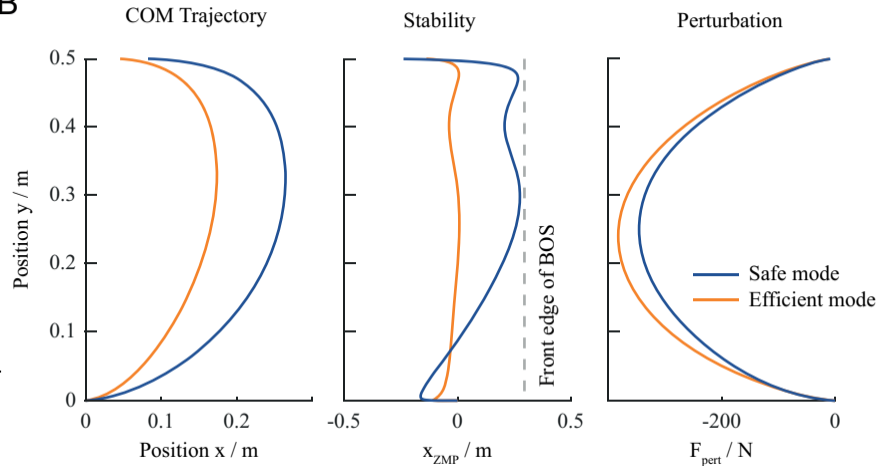

Supplement: S3 Fig — (A) Point-mass model used for mathematical optimization where we investigated how safety and movement efficiency influence the optimal trajectory of motion. Using a nonlinear programming solver, we determined optimal COM trajectories for (a) maximal safety where the centre of pressure (COP) was kept as far forward as possible, to maximise the postural stability with respect to the external perturbation acting in backward direction, and (b) for maximal movement efficiency where energy of motion was minimised. (B) The results of the optimization show that the optimal trajectories differ; if postural stability is maximised, the trajectory is arched more and hence both Trajectory Area and Initial Trajectory Area are larger than in the case when movement efficiency is maximised. Safe mode is shown in blue and efficient mode is shown in orange. The perturbation force of the efficient mode is larger than that of the safe mode due to a higher vertical velocity. (PDF) [file pcbi.1013089.s003.pdf]
