# Supplementary material for: Success-efficient/failure-safe strategy for hierarchical reinforcement motor learning
Source: PLoS Comput Biol. 2025 May 9;21(5):e1013089. doi: 10.1371/journal.pcbi.1013089 (PMC12121909; doi:10.1371/journal.pcbi.1013089)
Supplement: S5 Fig — Correlation for Trajectory Areas is illustrated in the upper pane, while the lower pane shows the correlation for Initial Trajectory Areas. Each bar represents a participant. The dark and light gray segments indicate the minimum and maximum correlation coefficients across the simulated Trajectory Areas and Initial Trajectory Areas, respectively. The mean correlation coefficients, along with their standard deviations, are shown using pink bars with error lines. The numbers above the bars indicate how many of the 59 simulation runs resulted in non-significant correlations (p < .05). Overall, the model captures the Trajectory Area patterns of participants to a high degree, as most participants exhibit significant correlations with the majority of the model-generated Trajectory Areas. However, Participant 7 shows the weakest correspondence, with 43 out of 59 simulated Trajectory Areas (59–16 = 43) still exhibiting significant correlations. In contrast, the lower pane shows that the model is less effective at capturing Initial Trajectory Area patterns for some participants. For example, participants 1, 6, and 20 exhibit a higher number of non-significant correlations, indicating that their Initial Trajectory Area patterns are not well captured by the simulation. (PDF) [file pcbi.1013089.s005.pdf]

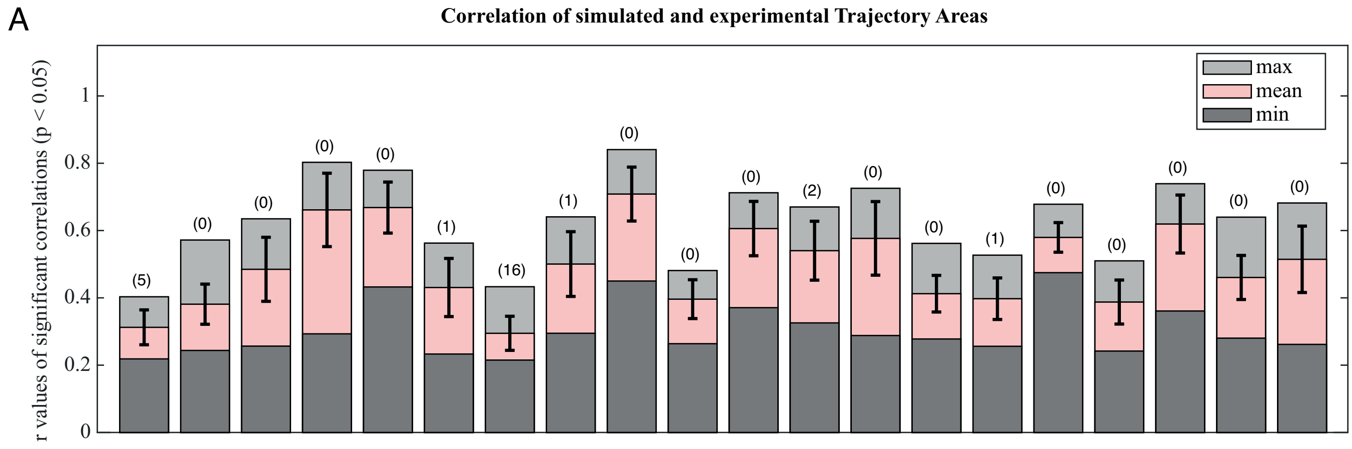

A
Correlation of simulated and experimental Trajectory Areas
r values of significant correlations (p < 0.05)
max
mean
min
(5)
(0)
(0)
(0)
(0)
(1)
(16)
(1)
(0)
(0)
(0)
(2)
(0)
(0)
(1)
(0)
(0)
(0)
(0)
(0)

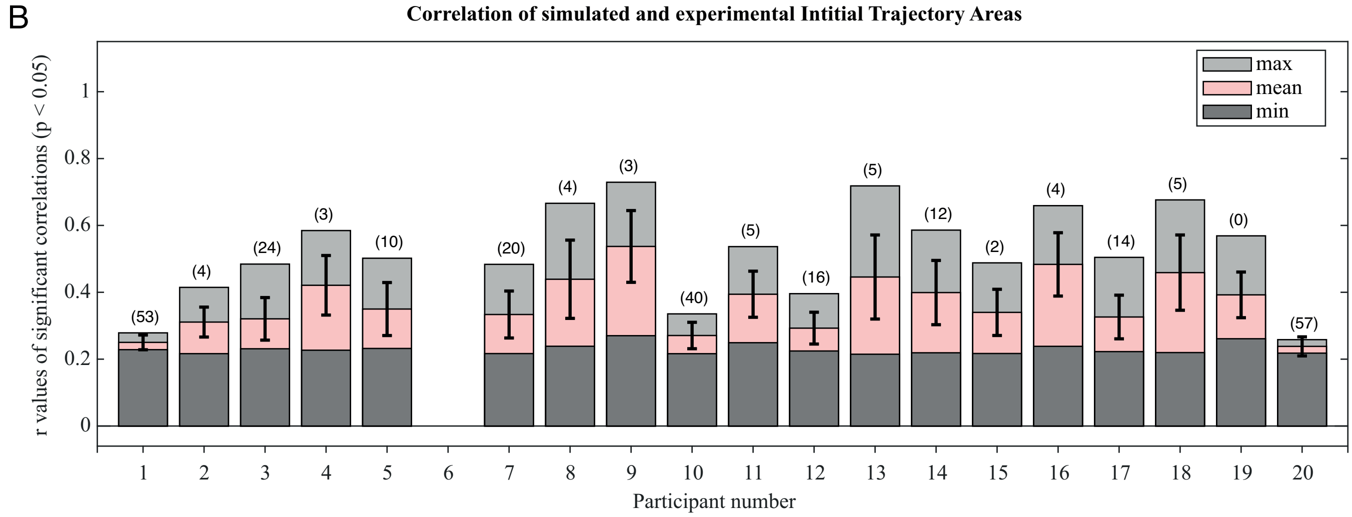

B
Correlation of simulated and experimental Intitial Trajectory Areas
r values of significant correlations (p < 0.05)
max
mean
min
(53)
(4)
(24)
(3)
(10)
(20)
(4)
(3)
(40)
(5)
(16)
(5)
(12)
(2)
(4)
(14)
(5)
(0)
(57)
Participant number
