# Supplementary material for: Success-efficient/failure-safe strategy for hierarchical reinforcement motor learning
Source: PLoS Comput Biol. 2025 May 9;21(5):e1013089. doi: 10.1371/journal.pcbi.1013089 (PMC12121909; doi:10.1371/journal.pcbi.1013089)
Supplement: S1 Table — Comparisons with significant differences are shown in bold. They are corrected for multiple comparisons using the Benjamini-Hochberg adjustment. *** < .001, ** < .01, * < .05. (PDF) [file pcbi.1013089.s006.pdf]

	Co-Contraction	Smoothness	Trajectory Area	Initial Trajectory Area	Number of Failed Trials
Number of Failed Trials	**$t = 3.40$ $p = .004$ ****	**$t = 4.61$ $p < .001$ *****	**$t = 2.19$ $p = .042$ ***	$t = 1.00$ $p = .328$	-
Initial Trajectory Area	**$t = 3.62$ $p = .002$ ****	**$t = 2.96$ $p = .010$ ***	**$t = 2.28$ $p = .034$ ***	-	-
Trajectory Area	$t = 1.99$ $p = .065$	$t = 1.82$ $p = .089$	-	-	-
Smoothness	$t = 1.44$ $p = .179$	-	-	-	-
Co-Contraction	-	-	-	-	-
