# Supplementary material for: Success-efficient/failure-safe strategy for hierarchical reinforcement motor learning
Source: PLoS Comput Biol. 2025 May 9;21(5):e1013089. doi: 10.1371/journal.pcbi.1013089 (PMC12121909; doi:10.1371/journal.pcbi.1013089)
Supplement: S1 Algorithm — (PDF) [file pcbi.1013089.s007.pdf]

E1. INITIALIZATION

i. $req\_succ = 60$: required #successful trials

ii. $conf_{\mu} = 50;\ conf_{\sigma} = 50$: #successful trials to gain task confidence

iii. $\rho = 1,\ \eta = 1,\ \gamma = 5$: cost baseline parameter $0 \le \rho \le 1$ and learning rates

iv. Define $clip(x) = max\ ((1, x)\ , -0.5))$: function to bound the advantages

v. $b = 0.025 \times [1\ 1\ 1\ 0\ 0\ 0]$: forward bias in action selection

vi. Set $\mu_0\ and\ \sigma_0 = 0.02$: RL initial action distribution

vii. $r_{last} = 0, trialc = 0, succ = 0, a_{mem} = [\ ]$: last trial success, #trials, #successes, good solution memory

viii. Let $\mu = \mu_0, \sigma = \sigma_0$ and generate $q, \dot{q}, \ddot{q}$ based on μ

ix. Execute trial with $q, \dot{q}, \ddot{q}$ and obtain costs $(J_{fall}\ , J_{safety}, J_{effort})$ and task success (r)

x. Sample *conf_succ_cnt* ~ $N(conf_{\mu}, conf_{\sigma})$: After how many successful trials the model reaches confidence (this is useful when emulating multi-subject experiments)
