# Supplementary material for: Success-efficient/failure-safe strategy for hierarchical reinforcement motor learning
Source: PLoS Comput Biol. 2025 May 9;21(5):e1013089. doi: 10.1371/journal.pcbi.1013089 (PMC12121909; doi:10.1371/journal.pcbi.1013089)
Supplement: S2 Algorithm — (PDF) [file pcbi.1013089.s008.pdf]

E2. RUN THE EXPERIMENT

1. Starting a new trial, increase trial counter: $trialc = trialc + 1$
2. If last trial was a failure and we have a good solution in memory then restore μ from it

   If $r_{last} == 0$ and $a_{mem} \neq$ [ ] then $\mu = \mu_{mem}$
3. Sample a from $\pi(a) \sim N(\mu + b(1 - r_{last}), \sigma)$ and apply parameter constraints
4. Generate $q, \dot{q}, \ddot{q}$ from a by using B-spline representation
5. Execute trial with $q, \dot{q}, \ddot{q}$ and get costs (J_{fall} , J_{safety}, J_{effort}), task success (r)
6. Let $succ = succ + r$ (keep track of the number of successful trails)
7. If $succ = conf\_succ\_cnt$ then let $conf = 1$ otherwise $conf = 0$
8. Compute cost advantages $Adv_x = clip\left(\underline{J}_x - J_x\right)$ for $x \in \{fail, safety, effort\}$
9. Update baselines $\underline{J}_x = (1 - \rho)\underline{J}_x + \rho J_x$ for $x \in \{fail, safety, effort\}$
10. EC control: As Adv_{ecol} return Adv_{fall} if failed; otherwise return Adv_{effort} if success and confident return else return Adv_{safety}

    $$Adv_{ecol} = (1 - r)Adv_{fail} + r\big((1 - conf)Adv_{safety} + \gamma conf Adv_{effort})\big)$$
11. Update the movement policy: $\mu = \mu + \eta(a - \mu)Adv_{ecol}$ and reduce exploration: $\sigma = 0.997\sigma$
12. $r_{last} = r$; if $r = 1$ (last trial success), update a_{mem} to keep the safest solution found so far
13. Use the execution experience to update the Inverse Dynamics Model
14. If $succ < req\_succ$ go to step 1 (do more trials until required number of successes is reached)
