# Supplementary material for: Success-efficient/failure-safe strategy for hierarchical reinforcement motor learning
Source: PLoS Comput Biol. 2025 May 9;21(5):e1013089. doi: 10.1371/journal.pcbi.1013089 (PMC12121909; doi:10.1371/journal.pcbi.1013089)
Supplement: S3 Algorithm — (PDF) [file pcbi.1013089.s009.pdf]

B1. INITIALIZATION

1. BufferSize = 600 : Data buffer size to use for learning the Inverse Dynamic Model (IDM)
2. BatchSize = 240 : Size of the data set used in batch learning
3. $\beta = 0.5$: Weight determining the effect of sample based local dynamics
4. $W = 0$ **:** Weight matrix determining the learned global inverse dynamics
5. Start with empty **X** and **Y** data buffers for (state and accelerations) and command respectively

B2. EXPERIENCE BUFFERING:

1. In each trial, store the state and accelerations $(q(t), \dot{q}(t), \ddot{q}(t))$ in **X;** the command $u(t)$ in **Y** as rows
2. If the number of rows > BufferSize start overwriting the stored data from the first rows of **X** and **Y**

B3. LEARNING:

1. Take a uniformly random row subset of **X** and **Y** with size BatchSize to form **X'** and **Y'**.
2. Apply a fixed nonlinear mapping (see B5) to the rows of **X'** to obtain: $\boldsymbol{Z} = \varphi(\mathbf{X}')$
3. Make the learning target an offset from the unperturbed dynamics $\Gamma(.)$: $\mathbf{V} = \mathbf{Y} - \Gamma(\boldsymbol{X})$
4. $\mathrm{W}_{loc} = \mathbf{Z}^{\dagger}\mathbf{V}$ where operator † indicates left pseudoinverse
5. $W = \beta W_{loc} + (1 - \beta)W$ (slowly shift the global IDM towards the computed sample-based local inverse dynamics model)

B4. PREDICTION

1. Given a state and a desired acceleration, $x = (q, \dot{q},\ \ddot{q}_{des})$, the predicted command is given by

$$u = \varphi(x)W + \Gamma(x)$$

B5. Additional Information for Algorithm 2

- $\varphi(x) = (x, sin\ sin\ (x_2)\ , x^2, x^3)$ where $x = [q_1, q_2, \dot{q}_1, \dot{q}_2, \ddot{q}_1, \ddot{q}_2]$ and powers are taken component-wise
