# Supplementary material for: Success-efficient/failure-safe strategy for hierarchical reinforcement motor learning
Source: PLoS Comput Biol. 2025 May 9;21(5):e1013089. doi: 10.1371/journal.pcbi.1013089 (PMC12121909; doi:10.1371/journal.pcbi.1013089)
Supplement: Attachment — Submitted filename: HiReL_response_to_reviewers.docx [file pcbi.1013089.s011.docx]

Dear Editor and Reviewers,

We sincerely thank you for your thoughtful and constructive comments. We greatly appreciate the time and effort invested in reviewing our manuscript. The feedback has been very helpful in improving the clarity and quality of the work. We have carefully addressed each comment and made the corresponding changes in the revised manuscript. Below, we provide a point-by-point response, with line numbers referring to the revised version without track changes. A tracked-changes version in the original manuscript style is also provided for easier comparison with the initial submission.

## Reviewer #1:

General:

Here the authors conducted a crouched to stand task, where participants were physically perturbed while attempting to stand. They analysed center of mass (COM) trajectory changes over time, including examining when the participants did make a corrective step (“fail”) or did not make a corrective step (“success”). They then modelled the movement behaviour using a hierarchical model that searches for the final movement goal using a win-stay/lose-shift algorithm, while considering feedforward and feedback (PD) control. The paper has several positive aspects, including well conducted experiments and the general idea of a hierarchal model are reasonable. However there are several larger concerns that relate to conflating co-contraction with feedback control, trial-level behaviour analyses, not considering movement velocity and base of support, and some model assumptions. Can the authors please use *continuous line numbers*, as well as responding in a point-by-point manner while directing the reviewer using line numbers to the location of all changes.

*We appreciate your valuable feedback on our work. Your comments have helped us refine the manuscript, and we have carefully addressed all your points. We hope the revised version meets your expectations. Please note that line number references below correspond to the revised manuscript without track changes.*

Major:

R1.1. Throughout the paper the authors conflate cocontraction and feedback gains. Cocontraction is generally thought to influence impedance control (see works by Etienne Burdet, David Franklin, and others), which is often used to handle unexpected perturbations. This is generally thought to be a different process than feedback control (e.g., Todorov, 2002; and work by several others). While it has been shown that co-cocontraction can elicit stronger feedback responses through automatic gain scaling (Pruszynski, J. A., Kurtzer, I., Lillicrap, T. P., & Scott, S. H. (2009). Temporal evolution of “automatic gain-scaling”. Journal of neurophysiology, 102(2), 992-1003.), impedance and feedback are generally considered different mechanisms that subserve motor control (Calalo et al., 2024: Journal of Neurophysiology, 129(4), 751-766).

While the authors have a behavioural assessment of impedance control through the dependent variable co-contraction, they do not have a behavioural assessment of feedback gains (i.e., short-latency, long-latency, or visuomotor). Accordingly, throughout the paper the authors should change the language from stating the co-contraction is a proxy of feedback control to impedance control, while also providing a paragraph or two on impedance and feedback control in the discussion. Further, since co-contraction is not a proxy of feedback control, there is a current disconnect with the computational model (see next comment).

*Thank you for your insightful remark. We fully agree that the relationship between the measure of co-contraction, the impedance control, and the feedback control is complex and not yet fully understood. In our study we used co-contraction as a means to assess the general contribution of feedback to motor control, without going into the specific details of impedance control and feedback control mechanisms. Our rationale behind linking co-contraction with feedback gains (rather than feedback control) is supported by evidence that feedback gains scale with muscle activity (Pruszynski et al., 2009) and that feedback responses are potentiated when muscles are co-contracted (Doemges & Rack, 1992; Akazawa et al., 1983; Krutky et al., 2010; Maurus et al., 2024). This approach is consistent with prior work, such as Heald et al. (2018), which similarly relied on this reasoning to interpret the interaction between co-contraction and feedback gains.*

*To ensure consistency, we carefully revised the text, replacing “feedback control gains” with “feedback gains” in two subsection titles. Furthermore, we added a Limitations section to the Discussion that examines the relationship between co-contraction, impedance control, and feedback control, incorporating several new references on this topic (lines 652 – 670). In this section, we also emphasized the limitations of our study and proposed potential directions for future research.*

R1.2. Computational modelling. The current computational model uses feedback gains on the positional and derivative terms (i.e., PD control). Yet the authors also have no true assessment of feedback gains in their behaviour results, making it difficult to reconcile the human behaviour with the implementation of the model. The paper does measure muscular co-contraction that is known to modulate impedance control, but there currently is no co-contraction and impedance control implemented in the model. The authors are encouraged to try to include impedance control within their model, so there is a more direct link between the model and behaviour. Alternatively, the authors will have to acknowledge this as a major limitation in the discussion.

*We fully agree that incorporating impedance control into the model would greatly enhance our understanding of how the brain modulates different control mechanisms. However, implementing impedance control would require a fundamentally different modeling approach, ideally involving the inclusion of individual muscles in the model. For this reason, we made a deliberate decision to omit impedance control during the initial planning of our study and reserve this for future research. While this limitation was briefly mentioned in the original manuscript (lines 449 - 453) we have now expanded on it in greater detail in the Limitations section of Discussion (lines 671 - 685).*

R1.3. The authors did well to perform a trial level analysis based on success and failure. Similar analyses have been done in sensorimotor adaptation, where one considers changes in behaviour following success or failure (Pekny, S. E., Izawa, J., & Shadmehr, R. (2015). Reward-dependent modulation of movement variability. Journal of Neuroscience, 35(9), 4015-4024.; Therrien, A. S., Wolpert, D. M., & Bastian, A. J. (2016). Effective reinforcement learning following cerebellar damage requires a balance between exploration and motor noise. Brain, 139(1), 101-114.; Roth, A. M., Calalo, J. A., Lokesh, R., Sullivan, S. R., Grill, S., Jeka, J. J., Carter. M.J., & Cashaback, J. G. (2023). Reinforcement-based processes actively regulate motor exploration along redundant solution manifolds. Proceedings of the Royal Society B, 290(2009), 20231475.; van der Kooij, K., & Smeets, J. B. (2019). Reward-based motor adaptation can generalize across actions. Journal of Experimental Psychology: Learning, Memory, and Cognition, 45(1), 71.;). However, the authors chose specific pairings and triplets of task outcome: Success-success and success-fail-success. While there is some value in looking at all combinations for the past three trials, examining the change in behaviour following a success or failure should be sufficient.

Thus, it is suggested that the authors examine the absolute change of each dependent measure following i) a success, or ii) a failure. An example is shown below for trajectory area as follows:

a) \delta(trajectory area) = trajectory area (trial n+1) - trajectory area (n); given trial n was a success)

b) \delta(trajectory area) = trajectory area (trial n+1) - trajectory area (n); given trial n was a failure)

Please also do this for the other variables, initial trajectory angle, co-contraction, smoothness. Also, there is no need to keep the somewhat arbitrary choice of S-S and S-F-S combinations. Finally, could the authors also include peak trajectory displacement. This is useful to think of in terms of whether a stability margin / base of support might be crossed.

*Thank you for the comment and the references provided. We apologize for not clearly explaining this in the original manuscript. We appreciate the suggestion to simplify the analysis and would like to take this opportunity to clarify key differences between our study and the referenced works (e.g., Pekny et al., 2015; Therrien et al., 2016; van der Kooij & Smeets, 2019; Roth et al., 2023), as well as most arm-reaching studies. These differences significantly influenced our decision to use the specific measures in our analysis, which were carefully chosen to address the specific requirements and objectives of our study.*

*The referenced studies investigate arm-reaching movements performed in constrained environments with minimal ecological consequences. These tasks are characterized by controlled conditions where the movement remains qualitatively similar irrespective of success or failure. In contrast, our study examines unrestrained whole-body squat-to-stand movements under significant force perturbations. Failures in our task lead to the critical ecological consequence of loss of postural balance and the necessity of a corrective step to avoid falling. This introduces a level of complexity and risk that is distinct from the failure outcomes typically observed in arm-reaching tasks. As a result, movements during failed trials in our study differ substantially from those during successful trials, as illustrated in Fig. 1C.*

*Applying the proposed analysis of absolute changes following success or failure to our task would predominantly capture the kinematic and dynamic differences between failed and successful trials, rather than isolating the adaptive contribution of the failure itself. Specifically, calculating the absolute change after a failed trial would mostly reflect the biomechanical difference between a failure-induced corrective movement and a successful trial, overshadowing the adaptive processes of interest. Comparing this to the absolute change after successful trials would not effectively unravel the contribution of failures to motor adaptation.*

*To address this, we analyzed the absolute changes between two consecutive successful movements with and without a failure in between. This approach allowed us to quantify the unique contribution of failed trials to motor adaptation by isolating how failures influence subsequent successful behavior. For clarity, we have amended the manuscript to explain this methodological decision and its rationale (lines 774 - 780). We hope this explanation clarifies our approach.*

*Thank you also for your suggestion to include peak trajectory displacement. We have added two diagrams, equivalent to Fig 1D and 1E, to the Supporting Information as S2 Fig and referenced them in the Results.*

R1.4. The authors have focused on position. However, again this is somewhat disconnected from past research in the field of biomechanics where it is well known that the base-of-support and CoM velocity are also important determinants of stability. For example, does it matter how far the CoM traverses as long as it is within the base of support? The authors have considered both velocity and base of support in their model. Perhaps I am mistaken, but I do not believe the authors are able to examine base of support behaviourally given the marker placements. However, they can examine CoM velocity. Can the authors please examine CoM velocity for successes and failures, and report these data in supplementary. Further, can they also show phase space figures by plotting position and velocity against one another, and also provide this information in supplementary material. These plots could also be separate for success and failure trials.

*Thank you for this comment. Below you can find the requested plots for individual subjects, including average velocity as a function of time, average velocity as a function of position in the y direction, and per-trial velocity as a function of position in the x direction. Black represents baseline trials, red represents failed trials, and green represents successful trials. The diagrams showing velocity with respect to position in the x direction are not averaged, as the motion in the x direction differs between successful/failed and unperturbed/perturbed trials. Averaging these data would mask meaningful differences and would not make sense. The velocity profiles are highly similar between successful and failed trials and do not provide new insights beyond what is already reported. The key difference between successes and failures appears in the x-position at equivalent velocities, which we have already demonstrated using position data. For this reason, we have not included these plots in the supplementary material. Nonetheless, we provide these diagrams for the reviewer’s consideration.*

Minor:

r1.1. The introduction is extremely broad, basically providing a brief summary of the many different subfields in sensorimotor neuroscience. However, the hypothesis revolves around the idea of the brain accounting for environmental risk / threats. Yet there is no literature review of this area, which is indeed very rich in the neuroeconomics literature including work done by Maloney and Landy, as well as some work done by Alaa Ahmed and more recently James Finley during gait. Also, the idea of fall risk also has an extremely large literature in gait and posture which have not been addressed in the introduction, including concepts such as base of support. In general, there should be a great deal of reworking/rewriting of the introduction which is currently does not sufficiently steer towards the hypothesis / question being tested.

*We appreciate the reviewer’s feedback. We have significantly revised the introduction to align it more directly with the study’s hypothesis. We have also incorporated relevant literature from neuroeconomics, including work by Maloney and Landy, as well as studies on risk-sensitive motor adaptation in gait and posture, such as those by Ahmed and Finley. Additionally, we have clarified the role of step placement in stability control, addressing its significance beyond our study’s classification of corrective steps as failures.*

r1.2. When “initial trajectory area” is first introduced, please specify the points of integration. Further, looking further down the manuscript the authors use a position distance of 2.5cm. What is the time that this takes on average? Voluntary feedback control can occur as early as 100 ms. Thus, this metric to capture feedforward control should be on average at 100ms or slightly less. Can the authors please comment and adjust their distance of 2.5cm if necessary.

*Indeed, we forgot to report the time which was 96.4 ± 7.0 ms (SD) from the start of the movement. We added this information where we first introduced the Initial Trajectory Area (lines 121 - 123) and in the Methods section (lines 748 - 751).*

r1.3. Can the authors please provide an equation when they introduce the term trajectory area. Note the equation is further below, but it is an important detail and currently unclear when reading the paper in a linear order. Further, please visually show this “area” by filling in the area being calculated with a sample trajectory, in a way that is more intuitive than currently shown in Figure 2. Intuitively one does not think of areas as negative. Perhaps just say “trajectory integral” instead of “trajectory area”. Then show a case with a trace where it is positive, and show a trace where it comes out negative. That will help the reader realize what the dependent measure means.

*Thank you for your suggestion. We have now included a definition of Trajectory Area and Initial Trajectory Area in the Results section, where the term is first introduced (lines 118 - 121). Moreover, we have included a figure S1 Fig to the Supporting Information that visually illustrates the computed area by filling in the region between the trajectory and the reference straight line. This figure includes examples of both positive and negative areas, making the concept more intuitive and helping readers better understand the dependent measure.*

r14. Here the authors frame a corrective step as a failure. It is often part of a successful gait repertoire during walking, which can be captured by optimal control. Will be useful to mention and cite works by Art Kuo and John Jeka on the role of step placement and stability in the discussion.

*We appreciate the reviewer’s suggestion. At the first mention of failure in the Results section (lines 113 - 115), we have added the clarification that while corrective steps are classified as failures within our study, they are widely recognized as an essential strategy for maintaining stability. We have also included references to support this perspective.*

r1.5. There has been a number of models in sensorimotor adaptation by Wolpert/Bastian and Cashaback on using reward/reinforcement to update motor plans, as well as the influence of movement variability following success and failure by Reza Shadmehr and Jeroen Smeets. Can the authors discuss similarities and differences of their approach to these classes of models and observed behaviours in the literature.

*Thank you for the constructive feedback. In the revision, we now discuss studies involving reinforcement feedback (reward), and movement variability in relation to our study (lines 520 - 540). In addition, for the comment on ‘movement variability following success and failure’ we have added a discussion spanning the lines 514 - 519 and 638 - 650.*

r1.6. please change the word “don’t” to “do not”.

*We have changed “don’t” to “do not” as requested.*

r1.7. There are several typos throughout the paper. Unfortunately, it is not possible to specify where they are because there are no line numbers. Can the authors please carefully reread this manuscript and correct each of the typographical errors.

*We carefully reviewed the entire manuscript with the help of a native English speaker and corrected all grammatical issues and typos. We hope that we have addressed all the errors and that none were accidentally overlooked.*

r1.8. There are run-on sentences throughout the manuscript. Please fix, which is readily accomplished by having one idea per sentence.

*We carefully reviewed the entire manuscript with the help of a native English speaker and corrected all instances of run-on sentences. We hope that none were accidentally overlooked.*

r1.9. Define “performance error”, “internal model” when first introduced.

“Performance error” was incorrect. We changed the two o*ccurrences in Introduction to “motor error.” Moreover, we defined the term “internal model” as you have suggested.*

r1.10. “normalised” suggests dividing by some value. I assume the authors meant they considered 84 trials before the catch trials? Also, is it not important to consider all the trials and not remove earlier trials where most of the learning takes place? Can the authors please comment.

*In our study, participants were required to complete 60 successful perturbed trials, but due to individual differences in learning rates, the total number of perturbed trials before the catch trial varied across participants (84.35 ± 11.28 on average). Importantly, no early trials were excluded from any analysis. To ensure comparability across participants, we normalized the number of perturbed trials to 84 - the average number of perturbed trials before the catch trial. This was achieved by scaling each participant’s trial numbers proportionally: specifically, for a subject with N perturbed trials, we computed the normalized trial index as (trial number / N) × 84. This approach retained all trials while aligning adaptation trajectories across participants, enabling meaningful averaging without omitting any data. We have addressed this concern by adding a clarification in the Methods section, explicitly stating how the normalization was performed (lines 724 - 727).*

r1.11. When first bringing up that the model adapted the movement plan, can you please specify exactly how this was implemented.

*Thank you for the suggestion. We updated the manuscript to specify that the model adapted the movement plan by reinforcement learning while also learning the novel dynamics created by the perturbation with an internal model using prediction error feedback (lines 345 - 346).*

r1.12. Can the authors please report in the supplementary the results of the co-contraction fitting when the 3 subjects that were removed were also included.

*Unfortunately, the data for those subjects was excessively noisy, making it practically unusable. As a result, we were unable to extract any meaningful muscle activations and did not proceed to the fitting stage.*

r1.13. Model. optimal feedback control argues against trajectory planning, but rather a more flexible response using feedback gains towards the final goal/target (see Cluff, T., & Scott, S. H. (2015). Apparent and actual trajectory control depend on the behavioral context in upper limb motor tasks. Journal of Neuroscience, 35(36), 12465-12476.). Can the authors please comment.

*It is true that theoretically optimal feedback control (OFC) can be used to solve any motor task without requiring an intermediate trajectory planning (TP) step. It is not a fully settled issue as to what extent the brain uses OFC and TP. There are strong opponents of the two views. We adopt a computational cost viewpoint and argue that pure OFC that requires a flat architecture for its solution is intractable for complex control problems. Without adhering fully to trajectory planning we adopt a general ‘motor plan’ representation that allows modular and hierarchical solutions to be adopted. This allows the CNS (and computers) to employ computationally lightweight solutions. Thus, with an ecological viewpoint we think that the CNS must have evolved to generate intermediate representations -which may not be the desired trajectory of robotics- for computational efficiency. In the revision we now state our viewpoint without rejecting OFC (lines 623 - 628).*

r1.14. Fig. 6. Can the authors use other colours than red / green, which is difficult to see for those that are colour blind.

*Thank you for pointing this out. We have adapted the red, green, blue, and orange (now pink) color shades to those in the Color Universal Design (CUD) palette (https://www.color-hex.com/color-palette/49436). This ensures that the figures are accessible and easily interpretable for individuals with color vision deficiencies.*

r1.15. Can the authors please look at the mean power frequency of the vastus medialis to see if it decreases over the course of the experiment, which will provide an indication on whether the participant was fatigued.

*We thank the reviewer for this suggestion. Upon examining the mean power frequency of the vastus medialis EMG, we found no decrease across trials, indicating no muscle fatigue. This aligns with our experimental design, which incorporated 30 s breaks between trials specifically to prevent fatigue. For clarity, a diagram illustrating the mean power frequency across trials is provided below.*

## Reviewer #2:

This paper integrates ecological factors (success vs. catastrophic failure) with a typical adaptation framework in which an established controller adjusts to an external perturbation. In a novel paradigm, participants here adapt to a force-field while performing squat-to-stand movements. In contrast to more typical adaptation tasks which involve arm reaching where failure is not catastrophic (participants may merely miss the reaching target), in this paradigm failure entails the ecologically-relevant danger of falling. Participants adapt to this force-field using a combination of both planning (which seems to be explicitly controlled) and implicit adaptation (which results in aftereffects even when participants are aware of the removal of the perturbation). Following failure, participants shift both their strategy and also show more overall learning. In addition, they tend to increase co-contraction. The paper presents a computational model which captures these effects.

This is a very interesting paper, with a well-designed behavioral experiment which is a welcome and ecologically-relevant addition to the repertoire of motor tasks used in the field. The modeling captures the behavioral findings well, and offers a framework to study the interaction between success/failure and sensorimotor learning, useful in generating further testable hypotheses. I have only a few questions before recommending acceptance.

*Thank you for your kind and encouraging feedback on our work. We have carefully addressed your questions and hope the revisions meet your expectations.*

R2.1 Figure 4C/D compare changes in smoothness and co-contraction. Something that limits the comparisons is that, failures are more likely early, than late in learning, when the participants haven’t reached asymptote yet. This could mean that S-F-S is more likely early in learning compared to S-S; and thus changes between the second and first S in S-F-S could represent both learning (because we are still far from asymptote) and response to the failure itself. It might be helpful to look at these analyses during asymptote learning, though I wouldn’t expect a substantial difference.

*We fully agree with your comment that S-F-S trials are more likely to occur early in adaptation, while S-S trials are more frequent later in adaptation, which could potentially affect our results. To address this, we have analyzed the data separately for the first and second halves of adaptation. The diagrams below illustrate this comparison, with the left column representing the first half and the right column representing the second half of adaptation, showing that the observed trends remain consistent across both phases of adaptation. Since the S-F-S condition is sparse, especially toward the end, an analysis based on asymptotic learning would not be faithful, and we have therefore omitted it.*

R2.2 Trials where adaptation was low / error was large are the ones more likely to be Failure trials. And, of course, participants respond more strongly to larger errors than smaller ones. Is there an effect of Failure (whether the participant had to perform a corrective movement) beyond what expected from the magnitude of the error during the Failure trial? One way to look at this might be, in the comparisons in Figs. 2BC/4CD, ask how does S-F-S compare to an S-S*-S trial, where S* is a trial with a large error similar to a Failure trial but where the participant did not do a corrective movement (i.e. a trial whereby trajectory area was similar to a failure trial, but was not a failure trial itself).

*We thank the reviewer for this insightful suggestion. To address this, we directly compared S-F-S trials, where corrective steps were taken (in blue), to S-S*-S trials, where S* trials had varying error magnitudes but no corrective steps (in orange). The diagram shows no systematic relationship between the magnitude of error in S* trials and subsequent adaptation. While one might expect participants to adapt more strongly following S* trials with the largest errors (i.e., those with the lowest trajectory area, TA < 110), the analysis shows that their adaptation was comparable to other successful trials and significantly smaller than after failed trials. This confirms that adaptation is primarily driven by the corrective movement itself rather than error magnitude alone. The grouping of S* trials was based on dividing the dataset into four groups according to TA, with TA < 110 representing successful trials roughly comparable to failed trials with TA of -32 ± 100 cm^2^.*

R2.3 What is the purpose of offering separate exponential fits for the learning curves for successful vs. failed trials (e.g. Fig. 3BC)? These are not separate learning curves or even separate components of learning; successful and failed trials are generated by the same learning curve. Is it that failed trials reveal a different component of learning? I’m not arguing against showing these trials separately as the authors do in Figure 1D or 3BC: it is interesting to show that even failed trials show substantial adaptation.

*The purpose of fitting separate exponential functions for successful and failed trials was to explicitly illustrate the distinct adaptation patterns observed in these two conditions. While both trial types are part of the same overall learning process, they exhibit different learning dynamics, particularly in terms of how trajectory adaptation evolves over time. By analyzing them separately, we were able to highlight key differences, such as the fact that Initial Trajectory Area only changed during failed trials, whereas Trajectory Area adapted in both successful and failed trials. This distinction supports our hypothesis that motor plan adaptation occurs primarily in response to failures, while overall adaptation continues throughout learning.*

R2.4 I do not understand why, in Figure 4C/D, “For each of the two conditions, we first calculated the sum of all changes during the perturbation block for each participant and then calculated the average values across all participants.” Why not estimate these changes as average per trial change for each participant (so the units would be the same as in 4A/B) and then calculate the average values across all participants? If taking the sum for each participant, then this will depend on how many S-F-S vs. S-S pairs are present for each participant. So somebody with many S-F-S pairs but a mediocre response per pair would appear to have a large response, whereas someone with few S-F-S pairs but a strong response per pair would appear to have a small response. Please explain/clarify if my understanding is incorrect.

*Thank you for your thoughtful comment. Our goal was to emphasize the overall contribution of S-F-S trials to adaptation as a whole. By summing the changes within each participant before averaging across participants, we ensured that the total adaptation could be decomposed into contributions from S-F-S and S-S pairs, illustrating that most adaptation is driven by S-F-S trials, despite their lower frequency. This approach highlights the cumulative effect of failures on adaptation, rather than emphasizing only the strength of individual S-F-S responses.*

*As you suggested, we have now also calculated the average per trial change for Initial Trajectory Area and Trajectory Area and included these values in the manuscript (results section Ecological control: Success-efficient/failure-safe learning strategy). This additional analysis provides an additional perspective, confirming that while S-F-S trials exhibit a stronger adaptation per trial, also their overall contribution to adaptation remains dominant despite being less frequent. For your information, here are also the diagrams showing the average per trial change of Initial Trajectory Area, Trajectory Area, Smoothness, and Co-Contraction.*

R2.5 Minor: it may be helpful to give an idea of the timing of these movements. For example, initial reach area is calculated at 2.5cm into movement – how much time has elapsed since movement onset? What is the total movement time? Figure 4E gives some idea of how long a movement is but, even there, the end of movement is not denoted.

*We appreciate the reviewer’s suggestion. The time elapsed at 2.5 cm into the movement was 96.4 ± 7.0 ms (SD) from movement onset, which we have now reported where Initial Trajectory Area is first introduced (lines 121 – 123) and in the Methods section (lines 748 - 751). Additionally, the total movement time was (Mean ± SEM) 1.16 ± 0.067 s, which has been added at the beginning of the Results section (lines 97 - 98).*

R2.6 Minor: In Figure 4, It would be interesting to also show baseline co-contraction/smoothness as a reference.

*Thank you for your suggestion. We have replaced Fig 4A and 4B with the updated version that includes the baseline values.*

## Reviewer #3:

The study by Jan Babic and colleagues introduces a novel computational motor control framework that integrates ecological fitness by alternating between success-driven efficiency and failure-driven safety, inspired by win-stay/lose-shift strategies. Through using an experimental paradigm on squat-to-stand movements under force perturbations to mimic an environmental threat, they demonstrate that humans adapt via hierarchical reinforcement learning mechanisms. They posited that humans effectively balance risk management and movement efficiency in real-world environments. Overall, this is an interesting study, particularly on the idea of integrating safety and the so-called efficiency of motor control, creating an ecologically relevant research topic.

While the manuscript is well written with good English, the authors must clear some questions before the manuscript can be accepted.

*Thank you for your thoughtful comments and positive feedback. We’ve addressed your questions and suggestions in the revised manuscript and hope the updates meet your expectations.*

R3.1. The authors employ some terminology across the manuscript. While the word safety seems self-explanatory, others may not be. For example, what do the authors mean by ‘efficiency’? In the later part of the manuscript, this was used together with the word energy efficiency after a series of successful movement productions.

*By movement efficiency we refer to the optimal use of muscles to perform movements with maximal output and minimal energy expenditure. We now use “movement efficiency” throughout the manuscript and added the definition to the Introduction (lines 77 - 78).*

R3.2. The authors proposed a model using an inverted pendulum and hierarchical reinforcement learning, but the model performance is missing. The authors should also be more explicit in quantifying the goodness of fit of their model to the behavioural (empirical) data, e.g. using R-squared or AIC. Alternatively, the authors can present alternative model comparisons, which are also missing.

*We thank the reviewer for the constructive comments. The computational model has been designed to capture the behavior of the human participants rather than maximizing performance (such as learning to stand up in the shortest time possible). Thus the performance of the model is actually how well it matches the learning patterns of the human subjects, which is exactly what is asked by the second comment of the reviewer. In the original manuscript we qualitatively compared the simulated model and human data by giving figures using the same conventions. But, we agree with the reviewer that having some quantitative measure for the model’s ability to capture human behavior would be an improving addition to the paper. Consequently, for the revision, we have performed a correlation analysis between human behavioral data and simulated data and included the results in the revised manuscript. In brief, the analysis showed that the model data has significant linear correlation between almost all subject’s data in terms of Trajectory Area. The power of the model to capture the Initial Trajectory Area patterns is found to be not as strong as the Trajectory Area patterns, but still more than the two thirds of the pair of human and model Initial Trajectory Area time series are found to show a significant linear correlation. The details of the new analysis are included in the revision spanning the lines 361 - 391 with an additional supplementary figure S6 Fig.*

R3.3. Ecological fitness: How is such framework proposed by the authors different from the exploration-exploitation behaviour in a pure reinforcement learning (reward-based) model of motor learning? Also, if the authors claim that failure is related to the safety mode. But why did the authors not compare S-S with F-F, instead of S-F-S? Didn't such F-F sequence provide a cleaner comparison with S-S?

*From the viewpoint of exploitation and exploration tradeoff in human decision making and reinforcement learning, in our model, successful motions encourage exploration of more efficient motions, whereas a failure switches to a safe policy which allows task completion without failure, which can be considered as exploitation. Our model offers a dynamically changing tradeoff between exploitation and exploration ensuring bodily well-being. In this sense it has more strict constraints compared to tasks where no real danger is present. In the literature it is documented that the tradeoff is dynamic and task specific (Shen & Dayan 2024; Averbeck 2016) but how the failures change the tradeoff is different from our setting, presumably due to risk of injury. Now we discuss these issues in the revision in the lines 507 - 519.*

*Thank you for your comment regarding the comparison of S-S with F-F instead of S-F-S. As you suggested, we have now calculated the F-F change for Initial Trajectory Area and Trajectory Area and included these values in the manuscript (results section Ecological control: Success-efficient/failure-safe learning strategy). This shows that F-F trials contribute to adaptation in a similar way as S-F-S trials, supporting the role of failures in shaping motor learning. For your reference, we have also provided diagrams below that illustrate the total change of Initial Trajectory Area, Trajectory Area, Smoothness, and Co-Contraction, where red bars highlight the total F-F change.*

Minor:

1. What is the function of the force plate (Figure 1A)? Any reason the authors did not incorporate any data from the Force Plate in the model?

*Without any particular reasons, the data from the force plate was not used in this study and we removed the label from Figure 1A.*

2. If a commercial product, please include the EMG sensor product/model name, together with the sampling rate. Also, this is supposed to be in sync with the Optotrak mocap.

*Indeed, we used commercially available EMG sensors from Biometrics Ltd. and recorded the muscle activities synchronous with the motion capture system with the sampling rate of 1000 Hz. We added these details to the Methods section of the manuscript (lines 765 - 766).*
