# Supplementary material for: Correction: Success-efficient/failure-safe strategy for hierarchical reinforcement motor learning
Source: PLoS Comput Biol. 2025 Sep 30;21(9):e1013537. doi: 10.1371/journal.pcbi.1013537 (PMC12483270; doi:10.1371/journal.pcbi.1013537)
Supplement: S4 Fig — Trial numbers and standard deviations of when the Number of Failed Trials, Trajectory Area, Initial Trajectory Area, Co-Contraction, and Smoothness reached their respective plateaus. (PDF) [file pcbi.1013537.s001.pdf]

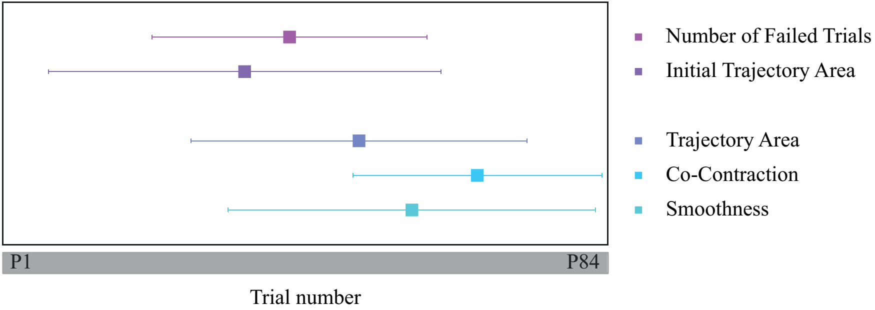

Number of Failed Trials
Initial Trajectory Area
Trajectory Area
Co-Contraction
Smoothness
P1
P84
Trial number
